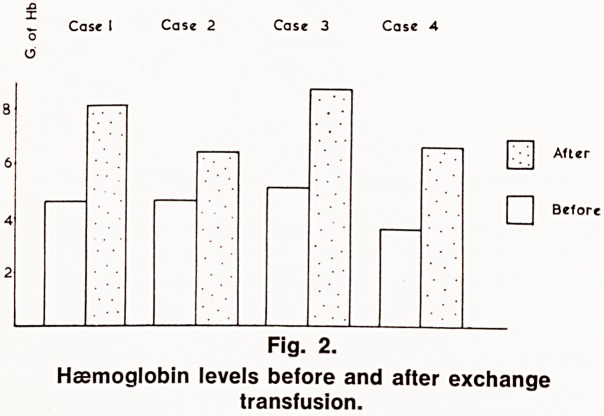# Exchange Transfusion in Severely Anæmic Adults with Heart Failure

**Published:** 1970-01

**Authors:** A. P. Cole

**Affiliations:** Medical Registrar, Cheltenham General Hospital


### Exchange Transfusion in Severely Anæmic Adults with Heart Failure

**A. P. Cole, D.C.H., M.R.C.P.**
Medical Registrar, Cheltenham General Hospital

The treatment of severe anæmia complicated by cardiac failure has usually been by a slow cautious transfusion as suggested by Marriott and Kekwick (1940), often combined with a diuretic. Alternatively hæmatopœtic agents may be given in deficiency states to allow physiological correction whilst the patient is resting in bed. The danger of the first course was pointed out by Sharpey Shafer (1945) when as little as 200 ml. of blood produced fatal pulmonary œdema, and the second course may be too slow in producing its effect. These patients therefore present the physician with a dilemma and can be a source of anxiety for a considerable time. In the face of bleeding the matter is even more difficult.

Walsh (1953) performed an exchange transfusion on a severely anæmic pregnant woman by simultaneous transfusion and venesections through arm veins and Fullerton and Turner (1962) did exchange transfusions on 115 severely anæmic pregnant women with five fatalities. It is interesting to note that these fatalities occurred in patients already in congestive failure.

The technique outlined here has been used on four patients, all elderly, whose hæmoglobin levels were raised rapidly without prejudicing their cardiac state. Only simple appartus was used, of the sort already in widespread use and the procedure can be easily performed at the bedside.

### TECHNIQUE

A large polythene Braun cannula is inserted into an ante-cubital vein. A Baxter giving set through which the blood will be transfused is attached. Into the rubber end of the Baxter set, a venesection needle is inserted. Venesection is achieved by applying a sphygmomanometer cuff with the transfusion stopped, and transfusion is achieved by letting down the cuff and withdrawing the venesection needle (See fig. 1).

**Fig 1.**
**Arrangement of apparatus for exchange transfusion.**

### CASES

**Case 1.** 83 year old woman. Three month history of tiredness, weakness and breathlessness on slight exertion. Both ankles had been swollen for two months. Initial Hb. 4.6 G/100 ml. Found to be drowsy, mucous membranes very pale, lemon tint to skin. Jugular venous pressure raised 5 cm, bilateral pitting ankle œdema, systolic murmur at the apex, bilateral basal crepitations. Marrow aspirate very cellular and showed megaloblastic erythropoiesis. Serum $B_{12}$ 343 micro-micrograms 100 ml, serum folic acid 3.0 millimicrograms 100 ml. ECG and chest x-rays within normal limits. Treated by bed rest, Cytamen and exchange transfusion using 600 ml. of concentrated cells (equivalent to 3 units packed together) taking 60 minutes. Result, Hb rose to 8G/100 ml. Immediate mental brightening. No change in cardiac signs. 48 hours later no signs of congestive cardiac failure found. Further good response to hæmatopoietic agents.

**Case 2.** A man of 70 had been passing melæna stools for 8 weeks. Attacks of giddiness and swollen ankles also for 8 weeks. JVP raised 2.5 cm, bilateral ankle œdema, cardiac enlargement, bilateral basal crepitations and 4 cm of hepatomegaly. Melæna stool on rectal examination. Initial Hb. 4.4G/100 ml. Subsequently a barium meal demonstrated an ulcer crater in the duodenal cap. He was treated with digoxin, Frusemide and bed rest and an exchange transfusion of 500 ml of concentrated cells (equivalent to the cells of 2 units packed together) which raised his hæmoglobin to 6.3 G/100 ml in 60 minutes. All signs of congestive cardiac failure settled in 72 hours. Post-gastric regime, oral iron and a very slow transfusion with 2 units of whole blood completed treatment. Discharged well.

**Case 3.** Woman of 74. History of angina of effort for 7 months. Admitted with a 4 day history of melæna stools. Epigastric pain after meals for 17 years. She experienced anginal pain on moving about in bed, was drowsy and exhibited "air hunger". Pale mucous membranes, apex beat displaced to the left, systolic murmur at apex conducted up into the neck. Bilateral basal crepitations. ECG showed left ventricular strain. Barium meal showed a large hiatus hernia with reflux. Initial Hb. 5.3G/100 ml. An exchange transfusion undertaken with $2\frac{1}{2}$ units of blood packed and given in 900 ml as concentrated cells in 90 minutes. Hb. rose to 8.5 G/100 ml. Immediate subjective improvement—became alert, lost angina and "air hunger". Treated with 4 more units whole blood by slow drip 3 days later. Discharged well on usual hiatus hernia regime.

**Case 4.** Woman of 81 with effort dyspnœa and ankle œdema for 3 weeks plus anorexia and nausea. Pale mucous membranes, lemon tint to skin, JVP raised 2.5 cm, bilateral pitting œdema to the knees, 4 cm of hepatomegaly; loss of vibration sense in both legs, muscle tenderness and bilateral extensor plantar responses. Initial Hb. 3.4G/100 ml. ECG showed ischæmic changes, cardiomegaly on chest x-ray and small left sided pleural effusion, macrocytic peripheral blood film, serum folic acid 4.0 millimicrograms 100 ml. Megaloblastic bone marrow. Treatment was with Cytamen, Frusemide and bed rest. Three days later still in congestive failure. Exchange transfusion raised Hb. from 3.4G to 6.4G/100 ml using 2 units of whole blood; congestive failure cleared slowly. On Cytamen had a reticulocyte response of 30%. Iron also added to treatment.

Fig. 2 shows the hæmoglobin levels before and after exchange transfusion in the four cases.

RESULTS

**Fig. 2.**
**Hæmoglobin levels before and after exchange transfusion.**

### DISCUSSION

Exchange transfusions in adults have been performed in severely anæmic pregnant women by simultaneously infusing and venesecting large volumes of blood. This was in otherwise healthy young women. Other workers have extracted the cells from the venesected blood and infused them also. In the present series exchange transfusions have been done on frail elderly patients in heart failure. Ony small quantities of blood were used and the circulating blood volume was not increased at all.

On the experience of these cases it is not possible to predict the results of exchange transfusion in a given case. One reason for this is that the total blood volume of chronically anæmic patients cannot be predicted on body weight alone as it tends to be diminished in these anæmic states. Clearly, though, the more anæmic the patient the better the response to exchange transfusion as the wastage of red blood cells by venesection is proportionally less. Also the use of concentrated infused blood produces better results and here a unit of blood is taken as that portion of the infused blood that remains after the subtraction of the anticoagulant added to it. In these four cases the anæmia was very severe and the maximum infused volume used was 900 ml. It is considered that in these cases the rise in oxygen-carrying capacity of the blood was sufficient to tide the patient over a critical phase so that ordinary transfusion became feasible in due course.

### SUMMARY

Four cases of severely anæmic patients with congestive cardiac failure were treated by exchange transfusion. Two had megaloblastic anæmias, one a peptic ulcer and one a hiatus hernia. Each was given not less than two units of blood in less than 90 minutes without producing a deterioration in the cardiac state, and in one case there was dramatic relief of angina. No special apparatus was used other than an ordinary venesection set, Baxter giving set, and Braun cannula. The best results are obtainable by infusing concentrated cells.

### Acknowledgement

The author wishes to thank Dr. N. R. Garden, Hæmatologist at the Cheltenham General Hospital for his assistance and invaluable help in preparing this article. He also thanks Dr. T. B. H. Haslett and Dr. R. G. Anderson for providing the cases.

### REFERENCES

**Fullerton, W. and Turner, G.** Exchange Transfusion in the Treatment of Severe Anaemia in Pregnancy. (1962) *Lancet* **2,** 570.

**Marriot, H. C. and Kekwick, A.** Volume and Rate in Blood Transfusion for the Relief of Anaemia (1940). *Brit. med. J.* **1,** 1043.

**Mollison, P. L.** Blood Transfusion in Clinical Medicine. Blackwell Scientific Publications. 1956. P. 595.

**Mollison, P. L., Veall, N. and Cutbush, Marie.** Red Cell Volume and Plasma Volume in Newborn Infants (1950). *Arch. Dis. Childh.* **25,** 242.

**Sharpey-Schafer, E. P.** Transfusion and the Anaemic Heart (1945). *Lancet* **2,** 296.

## Figures and Tables

**Fig 1. f1:**
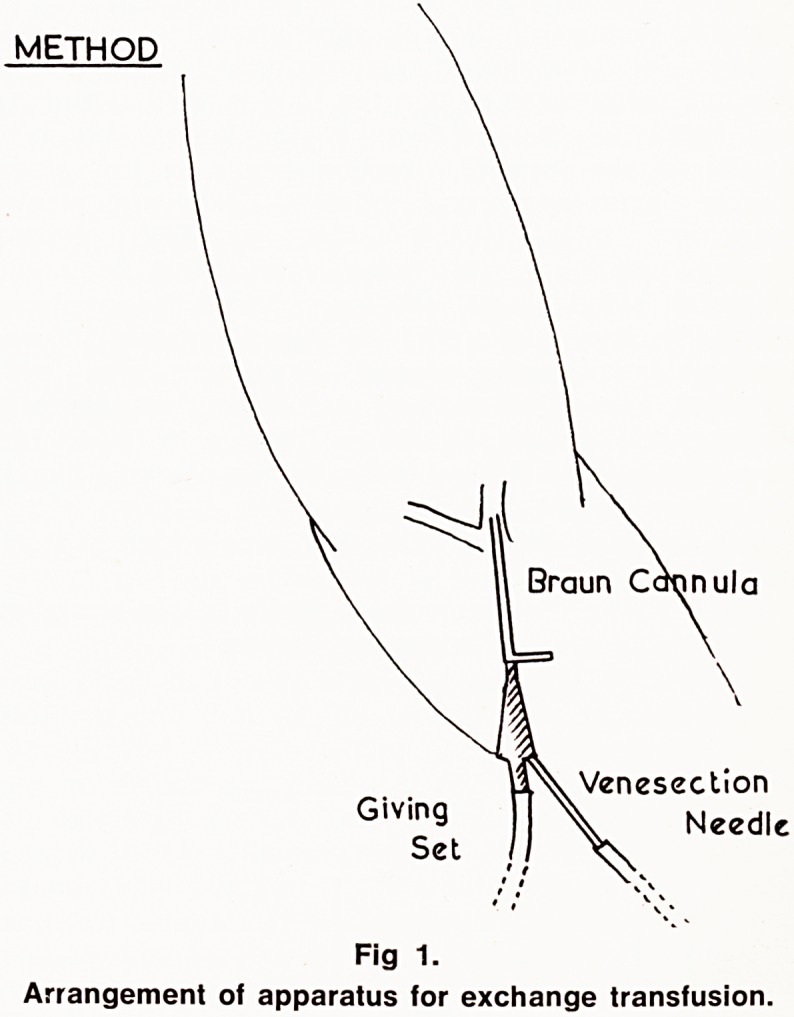


**Fig 2. f2:**